# CHSMiner: a GUI tool to identify chromosomal homologous segments

**DOI:** 10.1186/1748-7188-4-2

**Published:** 2009-01-15

**Authors:** Zhen Wang, Guohui Ding, Zhonghao Yu, Lei Liu, Yixue Li

**Affiliations:** 1 Key Lab of Systems Biology, Shanghai Institutes for Biological Sciences, Chinese Academy of Sciences, 320 Yueyang Road, Shanghai 200031, PR China; 2 Graduate School of the Chinese Academy of Sciences, Shanghai 200031, PR China; 3 College of Life Science & Biotechnology, Shanghai Jiao Tong University, Shanghai 200240, PR China; 4 Shanghai Centre for Bioinformation Technology, 100 Qinzhou Road, Shanghai 200235, PR China

## Abstract

**Background:**

The identification of chromosomal homologous segments (CHS) within and between genomes is essential for comparative genomics. Various processes including insertion/deletion and inversion could cause the degeneration of CHSs.

**Results:**

Here we present a Java software CHSMiner that detects CHSs based on shared gene content alone. It implements fast greedy search algorithm and rigorous statistical validation, and its friendly graphical interface allows interactive visualization of the results. We tested the software on both simulated and biological realistic data and compared its performance with similar existing software and data source.

**Conclusion:**

CHSMiner is characterized by its integrated workflow, fast speed and convenient usage. It will be useful for both experimentalists and bioinformaticians interested in the structure and evolution of genomes.

## Background

The identification of chromosomal homologous segments (CHSs) within and between genomes (known as paralogons and syntenies, respectively) is essential for comparative genomics. It can not only help evolutionary biologists to study genome evolution, such as genome duplication and rearrangement [1,2], but also help experimental biologists to transfer gene function information from one genome to another. Although extensive gene mutation, deletion, and insertion have made them not always obvious from primary sequences, chromosomal homology can still be revealed by a pair of segments sharing a group of homologous genes [3]. Most existing programs, including ADHoRe [4], FISH [5] and LineUp [6], look for CHSs based on the conservation of both gene content and order (colinearity). While the approach was sensitive enough for moderate divergence, it has been pointed out conserved gene order may be too strict for more ancient divergence [3], as inversion is another dominant force for the degeneration of CHSs. For example, the whole genome duplication in early vertebrate evolution can only be inferred by discarding gene order and considering gene content alone [7]. A pioneering implementation of this strategy was CloseUp [8], but some limitations still exist, especially with the rapid increase of genomic data. First, it used Monte Carlo simulation to estimate the statistical significance of identified CHSs, which might no longer be suitable for whole genome sequence analysis, as thousands of annotated genes would make it quite time-consuming. Second, previous tools were mainly developed for computational biologists, which restricted their wide use among experimental biologists.

In our recent project to build a paralog/paralogon database EPGD [9], we found it was very necessary to develop a new software that could overcome those weaknesses. Here, we publish it as a complete Java package named CHSMiner. Its core algorithm has been used to construct our database successfully and several improvements were added later as well. In short, it can not only fast identify and evaluate CHSs from whole genome comparison, but also provide a convenient graphical interface for end users to visualize the results.

## Implementation

### Fast greedy search algorithm

**Figure 1:**
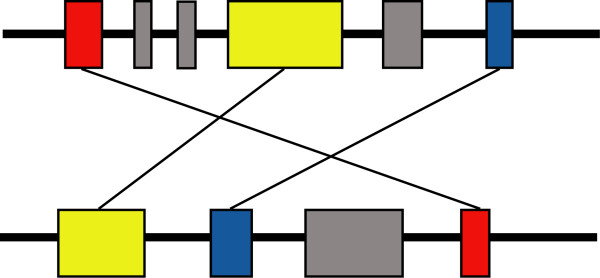
**Definition of CHS**. In this application, CHS is defined as two genomic regions that share a set of homologous (matched) genes, regardless of gene order and orientation. A limited number of unmatched genes can be allowed between two adjacent matched genes, but are restricted to be no more than a predefined constant, i.e. the maximal gap size.

CHSMiner defines CHSs based on shared gene content alone in order to fully exploit potential homology (Figure 1). Two major types of algorithms have been developed for the purpose in previous studies. One is based on the idea of bottom-up merging of smaller clusters (e.g. CloseUp [8]), and the other is based on top-down breaking the genomes (e.g. HomologyTeams [10]). We adopted the first strategy in CHSMiner because it was more widely used in relevant studies such as revealing ancient genome duplications [7]. Its procedure is also easier to understand: starting from two homologous genes, each at a different location, it looks for two other homologous genes that are each located within a prespecified distance from the former two ones. This process is iterated until no more additional pairs could be found [2,3]. The only important parameter that should be predefined is the maximal gap size (number of unmatched genes) allowed between two adjacent matched genes (Figure 1). Another advantage of the algorithm is that the greedy search has a fast computational speed because only a linear scan along a chromosome is needed.

### Formal statistical evaluation

Statistical test is necessary to reduce the false positive segments identified by the search algorithm. A null model commonly used for this purpose is based on randomization of gene order in the original genome [7]. If the CHS identified is impossible to form in the random genome, we can confirm gene associations within the segment. Previous programmes simulate the null model through permuting the genome repeatedly, but it is a time-consuming procedure. Fortunately, Hoberman et al. [11] have presented a mathematical treatment for max-gap gene clusters. On the basis of their conclusion, CHSMiner performs analytical test that can greatly reduce the computational burden. Specifically, we consider a set of *m *marked genes forms a cluster with maximal *g *insertions allowed between two adjacent genes. First, if we assume every family contains only one gene, the exact probability of observing the cluster in a random genome of *n *genes is [11]

$$P(n,m,g)=\frac{(n-m+1-(m-1)g/2){\left(g+1\right)}^{m-1}}{\left(\begin{array}{c} n\\ m \end{array}\right)}$$

Next, we consider the general case that a family contains more than one gene. We denote *F *= {*f*_1_, *f*_2_, ... *f*_*m*_}, where *f*_*j *_is the number of genes of the same family with gene *j *in the cluster. Then the probability above can be corrected as:

$$Q(n,m,g,F)=P(n,m,g)\prod_{j=1}^{m}f_{j}$$

Finally, we multiply the probabilities that the cluster is observed in both genomes for comparison, each with parameters (*n*_1_, *F*_1_) and (*n*_2_, *F*_2_):

*Q*(*n*_1_, *m*, *g*, *F*_1_)*Q*(*n*_2_, *m*, *g*, *F*_2_)

The value reflects the probability that a given CHS with maximal gap size *g *or smaller is observed in two independently and randomly ordered genomes. When the size of the CHS *m *is fixed, the smaller the maximal gap size is, the harder it can be observed. Therefore, the value can be treated as the *p*-value for the CHS. As a lot of CHSs should be assessed in whole genome comparison, we recommend an extra multiple test correction (e.g. Bonferroni correction) to the raw *p*-values in order to control false positive results.

### Java package and GUI for visualization

**Figure 2:**
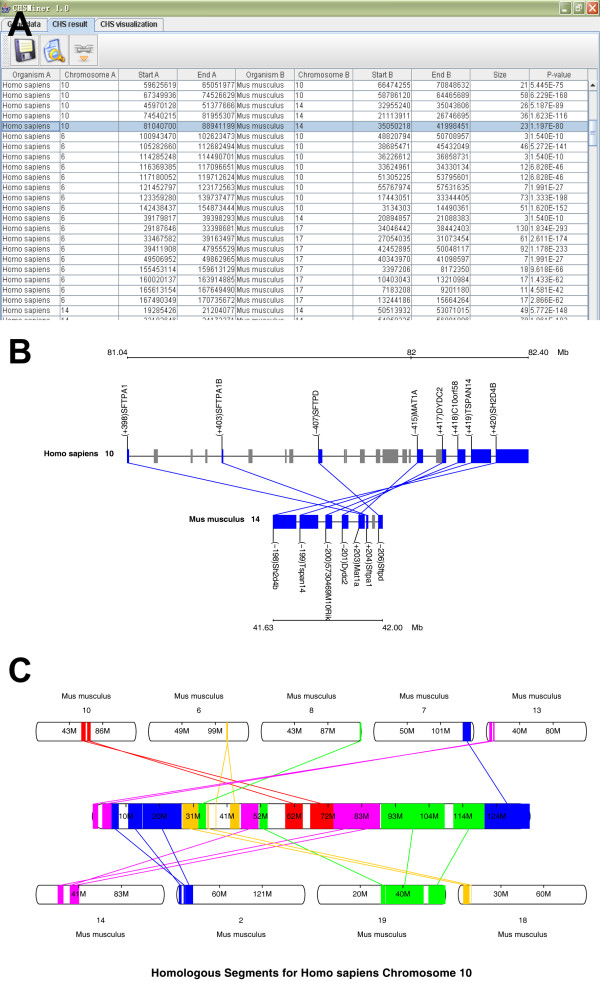
**Graphical display of CHS**. (A) CHSMiner organizes all identified CHSs as a table. It can generate two types of images for them. (B) Visualization of individual selected CHS, where homologous genes linked in the CHS are matched and labelled. (C) Visualization of a whole chromosomal pattern, where all homologous regions in a given chromosome are marked. The image is interactive and users can zoom in on a specific region.

CHSMiner is characterized by its graphical interface (Figure 2A) and several convenient features for end users include:

i. Automatic data download from Ensembl database [12] for well assembled genomes.

ii. Interactive operations and flexible parameter settings.

iii. Visual display of CHSs from an individual one to the whole genome pattern (Figure 2B, C).

iv. Useful graphic functions. The image can be saved as vector graph format for further edit.

The application was entirely written in Java and distributed as an executable jar package. It could run on any platform supporting Java Runtime Environment (1.5 or higher). Full source code and documents are also provided at our web site, and users can access them under GNU General Public License v2.0.

## Results and discussion

### Comparison on simulated data

**Figure 3:**
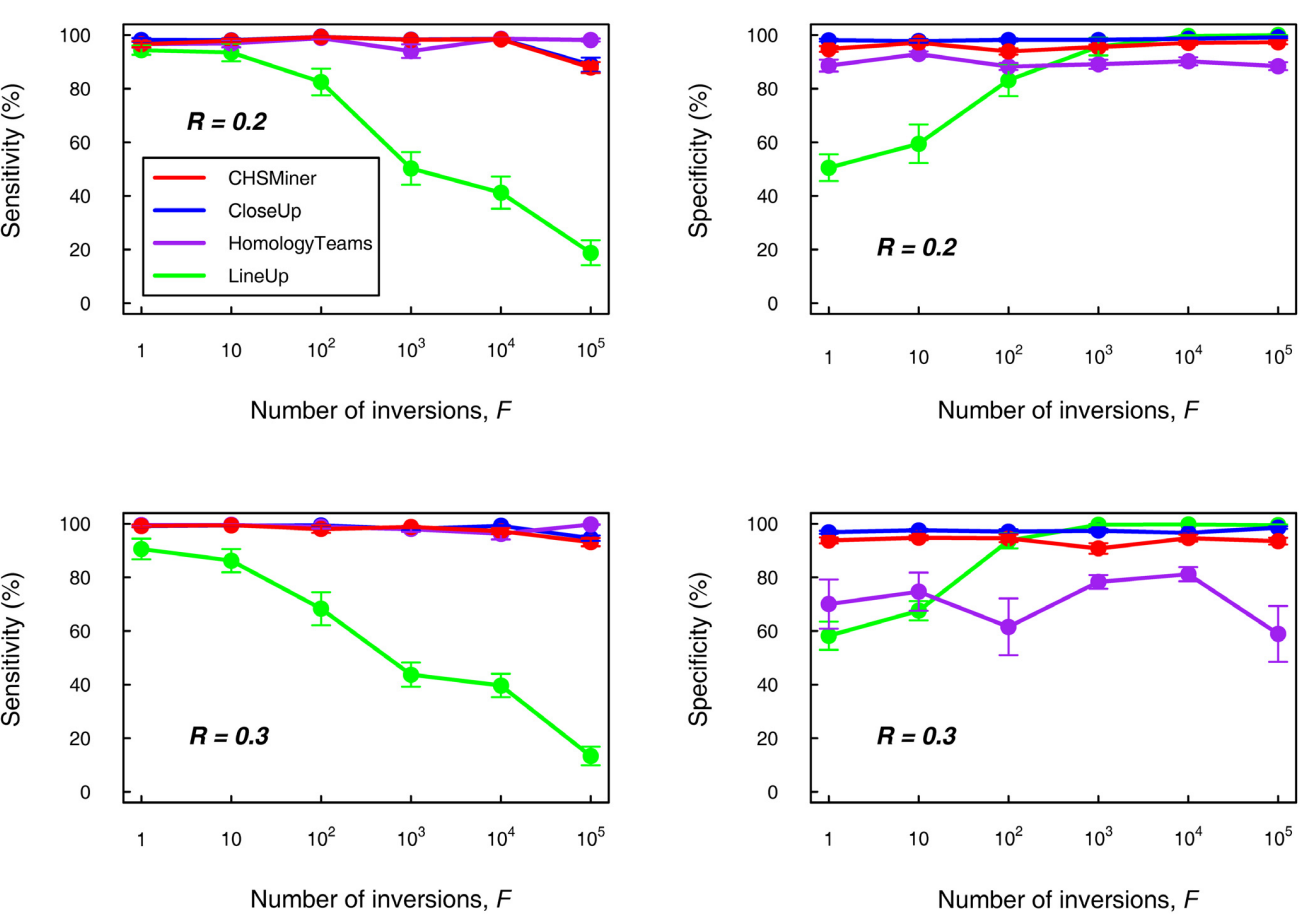
**Performance comparison on simulated data**. The extent of noise was controlled by the background similarity *R *and the number of inversions *F*. For each combination of *R *and *F*, 10 samples were simulated. Both sensitivity and specificity were calculated for the result of each sample (see Methods). The data point and error bar represent the mean value and the standard error of every percentage.

We used simulated data to compare CHSMiner with similar existing software as we can easily observe their performance by adjusting the extent of degeneration. We adopted the methods developed by Hampson et al. [8] to simulate two artificial chromosomes that contained a predefined CHS (see Methods). The fraction of conserved genes between the CHS was specified as 30%, which was approximate to biological realistic parameters [8]. Another two parameters were changed to adjust the noise against the CHS recognition: (1) background similarity *R*, and (2) the number of inversions *F*. Background similarity reflects extensive duplications and transpositions of individual genes [13]. In this analysis, *R *= 0.2 and 0.3 were chosen and *F *was varied between 1 and 10^5 ^to rearrange the gene order sufficiently. We compared CHSMiner with three other typical programmes for CHS detection, i.e. LineUp [6], CloseUp [8] and HomologyTeams [10] (Table 1). They were run on the simulated data set with the same parameter settings (see Methods). Both sensitivity and specificity were calculated for the results to evaluate the performance of the four programmes (Figure 3).

**Table 1:** Summary of the four programmes for comparison

Programme	CHS definition	Search algorithm	Statistical evaluation
CHSMiner	Gene content	Bottom-up	Analytical calculation
LineUp	Gene colinearity	Bottom-up	Monte Carlo simulation
CloseUp	Gene content	Bottom-up	Monte Carlo simulation
HomologyTeams	Gene content	Top-down	Not available

It is clear that the sensitivity of the algorithm based on colinearity will become gradually poor with the increase of inversions, whereas the algorithms based on gene content alone are quite robust to the disorders. HomologyTeams has the advantage of finding nonnested regions [14], but its gain of sensitivity is not evident until inversions are extremely frequent (>10^5^). In addition, as statistical validation is not implemented in HomologyTeams, its specificity will become quite lower when the background similarity is increased. CHSMiner and CloseUp can always have similar and satisfactory sensitivity and specificity for different *R *and *F*, suggesting that the analytical method of CHSMiner works as well as Monte Carlo simulation on empirical data. Nonetheless, CHSMiner is much faster than CloseUp. On a single Pentium processor CloseUp required more than one hour to run the simulated data set (1000 permutations for each CHS to get a reliable assessment), whereas CHSMiner took less than one minute. According to our experience, time is an important factor in genome comparison as we usually need to adjust parameters for the program. Thus, our tool greatly improves the efficiency and usability.

### Comparison on human-mouse synteny map

In order to show its performance on real biological data, we used CHSMiner to construct the synteny map for human and mouse. We downloaded homolog information from Ensembl database [12] and run the program with different maximal gap size. Each synteny detected was evaluated by corrected *p*-value (Bonferroni method) and only those smaller than 0.05 were preserved. The results were compared with the synteny map provided by Ensembl (release 47), which was generated from primary DNA sequence alignments [15].

We find our result is highly consistent with Ensembl map when the maximal gap size is equal to one gene (Table 2). There are 18753 orthologs present in Ensembl map, where 85% (15866) are found in our result. There are 3518 orthologs absent in Ensembl map, where 87% (3071) are not found in our result either. Furthermore, CHSMiner took only less than one minute to accomplish the analysis. Thus, our software has adequate power in both accuracy and efficiency to carry on large genome comparison.

**Table 2:** Number of orthologs covered by Ensembl synteny map and CHSMiner result

		CHSMiner result (by maximal gap size)				
			
		One gene		Five genes		Total
			
		Present	Absent	Present	Absent	
Ensembl synteny map	Present	15866	2887	18135	618	18753
	
	Absent	447	3071	1209	2309	3518

Total		16313	5958	19344	2927	

When we increase the maximal gap size to five genes, the coverage of detected syntenies will become larger (Table 2). Not only nearly all orthologs present in Ensembl map (18135 in 18753, 97%), but also an amount of ones absent in it (1209 in 3518, 34%) can now be discovered. The result does not change too much when the gap size is increased more (up to 30, data not shown). Since a strict statistical criterion has been applied for filtering, the newly obtained CHSs are less likely to be false positives. The reasonable interpretation is that those degraded CHSs can not be recognized from the primary sequence by the strategy of Ensembl. Therefore, CHSMiner is more flexible and can reveal more complete CHSs by selecting proper parameters.

## Conclusion

CHSMiner is designed to identify chromosomal homologous segments based on gene content alone, which enables it to discover highly degenerated homology. Compared with previous tools, it has at least three significant advantages: (1) it has comprised search algorithm, statistical validation and result display in a uniform platform; (2) it has improved both accuracy and efficiency; (3) its graphical and interactive interface allows it easy to use. We hope it will be helpful for biologists who are interested in the structure and evolution of genomes.

## Methods

### CHS simulation

First, two artificial chromosomes were created, each containing 1000 genes. The background similarity was simulated by assigning a gene to be the homolog of some other gene with probability *R*, regardless of their locations. Then the middle 20% of the two chromosomes were specified as a known CHS. Within the region, a gene in one chromosome would have a corresponding homolog in the other chromosome with probability 0.3. Finally, the inversions were simulated by exchanging two randomly chosen neighbouring gene pairs.

### Software comparison

All the four software packages were tested on the simulated data set with the same parameter settings, i.e. the gap size should be less than 20 genes and each CHS should have at least 3 matched genes. LineUp was run with inversions forbidden. If statistical test was available, each CHS detected was further assessed by corrected *p*-value (Bonferroni method) and only those smaller than 0.05 were preserved. The sensitivity was calculated as *P*/*TP*, where *TP *was the number of genes in the predefined CHS (*TP *= 200) and *P *was the number detected among them. The specificity was calculated as *N*/*TN*, where *TN *was the number of genes not in the predefined CHS (*TN *= 800) and *N *was the number remaining undetected in *TN*.

## Availability and requirements

Project name: CHSMiner

Project home page: 

Operating system(s): Platform independent

Programming language: Java

Other requirements: JRE 1.5 or higher

License: GNU GPL

## Competing interests

The authors declare that they have no competing interests.

## Authors' contributions

ZW designed the software, implemented the algorithm and drafted the manuscript. GD conceived of the software, and participated in its design. ZY participated in the discussion of biological significances. LL and YL revised the manuscript. All authors read and approved the final manuscript.
